# MicroRNA signatures predict oestrogen receptor, progesterone receptor and HER2/*neu *receptor status in breast cancer

**DOI:** 10.1186/bcr2257

**Published:** 2009-05-11

**Authors:** Aoife J Lowery, Nicola Miller, Amanda Devaney, Roisin E McNeill, Pamela A Davoren, Christophe Lemetre, Vladimir Benes, Sabine Schmidt, Jonathon Blake, Graham Ball, Michael J Kerin

**Affiliations:** 1 Department of Surgery, Clinical Science Institute, University Hospital/National University of Ireland Galway, Galway, Ireland; 2 John Van Geest Cancer Research Centre, School of Science & Technology, Nottingham Trent University, Clifton Campus, Clifton Lane, Nottingham NG11 8NS, UK; 3 European Molecular Biology Laboratory, Meyerhofstrasse 1, 69117 Heidelberg, Germany

## Abstract

**Introduction:**

Breast cancer is a heterogeneous disease encompassing a number of phenotypically diverse tumours. Expression levels of the oestrogen, progesterone and HER2/*neu *receptors which characterize clinically distinct breast tumours have been shown to change during disease progression and in response to systemic therapies. Mi(cro)RNAs play critical roles in diverse biological processes and are aberrantly expressed in several human neoplasms including breast cancer, where they function as regulators of tumour behaviour and progression. The aims of this study were to identify miRNA signatures that accurately predict the oestrogen receptor (ER), progesterone receptor (PR) and HER2/*neu *receptor status of breast cancer patients to provide insight into the regulation of breast cancer phenotypes and progression.

**Methods:**

Expression profiling of 453 miRNAs was performed in 29 early-stage breast cancer specimens. miRNA signatures associated with ER, PR and HER2/*neu *status were generated using artificial neural networks (ANN), and expression of specific miRNAs was validated using RQ-PCR.

**Results:**

Stepwise ANN analysis identified predictive miRNA signatures corresponding with oestrogen (*miR-342*, *miR-299*, *miR-217*, *miR-190*, *miR-135b*, *miR-218*), progesterone (*miR-520g*, *miR-377*, *miR-527-518a*, *miR-520f-520c*) and HER2/*neu *(*miR-520d*, *miR-181c*, *miR-302c*, *miR-376b*, *miR-30e*) receptor status. *MiR-342 *and *miR-520g *expression was further analysed in 95 breast tumours. *MiR-342 *expression was highest in ER and HER2/*neu*-positive luminal B tumours and lowest in triple-negative tumours. *MiR-520g *expression was elevated in ER and PR-negative tumours.

**Conclusions:**

This study demonstrates that ANN analysis reliably identifies biologically relevant miRNAs associated with specific breast cancer phenotypes. The association of specific miRNAs with ER, PR and HER2/*neu *status indicates a role for these miRNAs in disease classification of breast cancer. Decreased expression of *miR-342 *in the therapeutically challenging triple-negative breast tumours, increased *miR-342 *expression in the luminal B tumours, and downregulated *miR-520g *in ER and PR-positive tumours indicates that not only is dysregulated miRNA expression a marker for poorer prognosis breast cancer, but that it could also present an attractive target for therapeutic intervention.

## Introduction

Breast cancer is a heterogeneous disease that encompasses a range of phenotypically distinct tumour types. Underlying this heterogeneity is a spectrum of molecular alterations and initiating events that manifest clinically through a diversity of disease presentations and outcomes. Novel therapeutic strategies are increasingly being investigated and implemented, but unpredictable response and the development of resistance to adjuvant therapy remain major challenges in the clinical management of breast cancer patients.

The key to optimizing and targeting therapy lies in a more complete understanding of the complex molecular interactions that underlie breast cancer and contribute to its heterogeneous nature. Breast-cancer-related genes have been extensively investigated, largely through the development of high-throughput array-based gene expression profiling platforms. The substantial datasets that have ensued have enabled us to decipher in depth some of the molecular intricacies associated with breast cancer, and have expanded our knowledge of the genetic pathways associated with breast carcinogenesis, resulting in classification systems predictive of outcome [1,2].

Breast tumours can now be classified into major subtypes on the basis of gene expression – luminal, v-erb-b2 erythroblastic leukaemia viral oncogene homolog 2 receptors (HER2/*neu*) overexpressing and basal like – and further analysis has identified additional subtypes within the original subgroups [3]. The expression of specific genes such as the oestrogen receptors (ERs) and HER2/*neu *are indicative of outcome in breast cancer patients, and the clinically relevant subgroupings are based broadly on ER/progesterone receptor (PR)/HER2/*neu *status. The ability to classify breast cancers in this manner has obvious beneficial implications for the development of targeted therapies; multigene prognostic and predictive tests have been developed, have been commercialized and have become established as tools in breast cancer diagnostics [4], although as yet there is little knowledge regarding the precise regulation of these genes and receptors.

MicroRNAs (miRNAs) are short (~22 bp), single-stranded, noncoding RNAs that have recently been recognized as a highly abundant class of regulatory molecules. They are thought to regulate up to one-third of the human genome via sequence-specific regulation of post-transcriptional gene expression by targeting mRNAs for cleavage or translational repression [5]. miRNAs have recently been identified as key players in cellular processes including self-renewal, differentiation, growth and death [6], all of which are dysregulated in carcinogenesis. There is increasing evidence to suggest that miRNAs may be responsible for a large proportion of breast cancer heterogeneity. A number of miRNAs have been shown to be dysregulated in breast cancer [7-10], and specific miRNAs functioning as regulators of tumorigenicity, invasion and metastasis have been identified [11-14]. Furthermore, miRNA regulation of ER and HER2/*neu*, known to be of prognostic significance in breast cancer, has been demonstrated [15,16]. As each miRNA can target up to 200 mRNA sequences, and mRNAs can have multiple miRNA target sites [5], it is probable that further miRNA regulators of these genes remain to be determined.

Expression profiling of miRNA to classify breast tumours according to clinicopathological variables currently used to predict disease progression is of particular interest. Firstly, profiling highlights the potential to identify novel prognostic indicators, which may contribute to improved selection of patients for adjuvant therapy. This approach has already shown promise with genomic signatures [2], and miRNA profiles appear to have superior accuracy to mRNA profiling [17]. Furthermore, the identification of miRNAs with regulatory roles in clinically distinct breast tumour samples could identify novel targets for therapeutic manipulation.

Despite its apparent clinical application, microarray technology remains deficient with regard to its translation into routine clinical practice. There has been little overlap between the breast cancer gene sets, leading to questions regarding their biological significance and reproducibility [18]. Array technology is highly dependent on bioinformatics, mathematics and statistics to produce biologically relevant results. The generation of high-complexity microarray data has necessitated the development of novel data analysis methodologies that can cope with data of this nonlinear and highly dimensional nature. Current conventional methods such as hierarchical clustering have shown limitations for the modelling and analysis of high-dimensionality data [19].

Artificial neural networks (ANNs) are a form of artificial intelligence that can learn to predict, through modelling, answers to particular questions in complex data. The models produced by ANNs have been shown to have the ability to predict well for unseen data and have the ability to cope with complexity and nonlinearity within the dataset [20,21]; these features of ANNs means they have the potential to identify and model patterns in this type of data to address a particular question. ANNs are therefore able to determine patterns or features (for example, in genes or proteins) within a dataset that can discriminate between subgroups of a clinical population (for example, disease and control), or disease grades [22]. Indeed, this discrimination has been previously demonstrated in different tumour types [22,23]. These patterns can combine into a fingerprint that can accurately predict the subgroups.

Our aims in the present study were to identify miRNA signatures using ANNs that accurately predict the ER, PR and HER2/*neu *status of breast cancer patients, thus identifying potential biologically relevant miRNAs and providing further insight into breast cancer aetiology and regulation.

## Materials and methods

### Patients and samples

Breast tumour specimens were obtained from patients during primary curative resection at Galway University Hospital, Galway, Ireland. Matched tumour-associated normal breast tissue was also obtained from a subset of these patients where possible. Following excision, tissue samples were immediately snap-frozen in liquid nitrogen and stored at -80°C until RNA extraction. Prior written and informed consent was obtained from each patient and the study was approved by the ethics review board of Galway University Hospital. The initial cohort for microarray analysis consisted of 29 early-stage, invasive ductal carcinoma breast tumour specimens. A larger cohort of fresh-frozen breast tumour (n = 95) and tumour-associated normal breast tissue (n = 17) specimens was used for validation and further analysis of selected miRNAs. Clinical and pathological data relating to the clinical samples are presented in Tables 1 and 2.

**Table 1:** Clinical and pathological data for breast tumours analysed by microarray

Number	ID	Age (years)	Tsize (mm)	Lymph node status	Grade	UICC stage	ER	PR	HER2/*neu*	Subtype
1	52	49	23	Negative	1	2A	P	P	N	Luminal A
2	53	52	30	Negative	3	2A	N	N	P	Her2 overexpressing
3	54	57	45	Negative	3	2A	N	N	P	Her2 overexpressing
4	56	51	21	Negative	3	2A	P	P	N	Luminal A
5	58	68	15	Negative	3	1	P	N	N	Luminal A
6	59	42	22	Negative	3	2A	N	N	N	Triple negative
7	60	54	26	Negative	3	2A	N	P	N	Luminal A
8	61	35	22	Negative	3	2A	P	P	N	Luminal A
9	62	50	16	Negative	3	1	N	N	N	Triple negative
10	63	49	25	Negative	2	2A	N	N	N	Triple negative
11	64	59	20	Negative	3	1	N	P	N	Luminal A
12	65	58	22	Negative	3	2A	P	P	N	Luminal A
13	66	58	18	Negative	1	1	N	P	P	Her2 overexpressing
14	67	66	22	Negative	3	2A	P	P	N	Luminal A
15	94	56	17	Negative	1	1	N	N	N	Triple negative
16	95	48	30	Negative	3	2A	N	N	P	Her2 overexpressing
17	96	60	26	Negative	3	2A	P	P	N	Luminal A
18	97	56	29	Negative	2	2A	P	P	N	Luminal A
19	98	50	3	Negative	2	1	P	P	N	Luminal A
20	99	40	7	Negative	1	1	P	P	N	Luminal A
21	100	40	6	Negative	2	1	P	P	N	Luminal A
22	101	58	35	Negative	2	2A	P	P	N	Luminal A
23	102	64	34	Negative	3	2A	P	P	N	Luminal A
24	103	66	26	Negative	1	2A	P	P	N	Luminal A
25	104	84	16	Negative	2	1	N	P	N	Luminal A
26	105	57	7	Negative	3	1	N	P	N	Luminal A
27	106	68	35	Negative	3	2A	P	P	N	Luminal A
28	107	40	20	Negative	2	1	P	P	P	Luminal B
29	108	49	35	Negative	3	2A	N	N	N	Triple negative

ER, oestrogen receptor; HER2/*neu*, v-erb-b2 erythroblastic leukaemia viral oncogene homolog 2 receptors; ID, identification; N, negative confirmed; PR, progesterone receptor; P, positive confirmed; Tsize, Tumour size in mm; UICC, stage of breast tumour according to the international union against cancer staging criteria.

**Table 2:** Clinical and pathological data for breast tumours in the independent validation cohort

Breast cancer clinicopathological characteristic	Number of patients (n = 95)
Median (interquartile range) tumour size (mm)	23.5 (17.75 to 35.0)
Histologic subtype	
Invasive ductal	80
Invasive lobular	13
Colloid/mucinous	1
Tubular	1
Tumour-associated normal	17
Intrinsic subtype	
Luminal A (ER/PR^+^, HER2/*neu*^-^)	47
Luminal B (ER/PR^+^, HER2/*neu*^+^)	21
Her2 overexpressing (ER^-^, PR^-^, HER2/*neu*^+^)	11
Triple-negative (ER^-^, PR^-^, HER2/*neu*^-^)	11
Missing data	5
Grade	
1	14
2	26
3	53
Missing data	2
Nodal status	
Node-negative	50
N1	17
N2	17
N3	11
Oestrogen receptor status	
Positive	62
Negative	32
Missing data	1
Progesterone receptor status	
Positive	58
Negative	33
Missing data	4
Her2/*neu *status	
Positive	32
Negative	59
Missing data	4
UICC stage	
Stage 1	23
Stage 2a	29
Stage 2b	8
Stage 3a	14
Stage 3b	4
Stage 3c	8
Stage 4	9

ER, oestrogen receptor; HER2/*neu*, v-erb-b2 erythroblastic leukaemia viral oncogene homolog 2 receptors; PR, progesterone receptor; UICC, stage of breast tumour according to the international union against cancer staging criteria.

The ER, PR and HER2/*neu *status of the patients was determined by immunohistochemistry on formalin-fixed, paraffin-embedded sections of clinical specimens as part of routine pathology to guide clinical decision-making regarding adjuvant therapy. Immunohistochemistry was performed using a rabbit monoclonal antihuman ER antibody (clone SP1; Dako, Cambridgeshire, UK) and a polyclonal rabbit antihuman PR antibody (Dako). The Allred scoring method was used for expression scoring of ER and PR based on proportion and intensity. In brief, the proportion score represented the estimated percentage of tumour cells staining positive (0 = 0%; 1 = 1%; 2 = 1 to 10%; 3 = 10 to 33%; 4 = 33 to 66%; 5 = > 67%), and the intensity of staining was scored as follows: 1 = weakly positive; 2 = moderately positive; 3 = strongly positive. The total score was derived from the following equation, a score of 0 being negative and a score of 2 to 8 being positive:



Membranous staining was scored for HER2/*neu *according to the HercepTest (Dako) as follows: 0 = negative; 1 = weak incomplete membranous staining of > 10% cells (negative); 2 = weak – moderate complete membranous staining of > 10% of cells (equivocal-fluorescence *in situ *hybridization was used to assess amplification in these cases); 3 = strong complete membranous staining of > 30% of cells (positive).

### miRNA microarray

#### RNA extraction

Depending on whether samples were destined for microarray or RQ-PCR analysis, slightly modified RNA extraction methods were employed. For the microarray experiment, total RNA was required. Breast tumour tissue (50 to 100 mg) was homogenized using a bench-top homogenizer (Polytron^® ^PT1600E; Kinematica AG, Littau-Luzem, Switzerland) in 1 ml QIAzol lysis reagent (Qiagen, Crawley, UK). Total RNA was isolated from homogenized breast tissue using the RNeasy^® ^Tissue Mini Kit (Qiagen) according to the manufacturer's instructions. For RQ-PCR, miRNA was selectively isolated from approximately 100 mg tissue.

Large RNA fractions (> 200 nucleotides) and small RNA fractions (< 200 nucleotides) were isolated separately using the RNeasy Plus Mini Kit and RNeasy MinElute^® ^Cleanup Kit (Qiagen) according to the supplementary protocol: purification of miRNA from animal cells. The concentration and purity of total RNA were assessed using a NanoDrop™ 1000 spectrophotometer (Nanodrop Technologies, Wilmington, DE, USA). RNA integrity was assessed using the RNA 6000 Nano LabChip Series II Assay with the 2100 Bioanalyzer System (Agilent Technologies, Palo Alto, CA, USA). Electropherograms and gel-like images were evaluated using the Agilent 2100 Expert software (version B.02.03; Agilent Technologies, Palo Alto, CA, USA), which generated the RNA integrity number to ensure that only RNA of good integrity was used in these experiments (RNA integrity number range, 7.6 to 9.5). The miRNA concentration and purity were also assessed by NanoDrop™ 1000 spectrophotometry. Small miRNA-enriched fractions were analysed using the Small RNA Assay on the Agilent 2100 Bioanalyzer.

#### RNA labelling and microarray hybridization

Total RNA was Cy-dye labelled and hybridized on miRNA microarray chips as previously described [24]. Briefly, 5.5 μg total RNA was 3' ligated to Cy dye-linked 2'-deoxyuridine-5'-triphosphate using T4 RNA ligase (catalogue number 2141; Ambion, Woodward, Austin, TX, USA), in the presence of RNase inhibitor (catalogue number 2682; Ambion, Woodward, Austin, TX, USA), ATP (Grade I, catalogue number A2383-1G; Sigma-Aldrich Corp. St. Louis, MO, USA), and polyethylene glycol 50% aqueous solution (PEG 6000, catalogue number 81304; Fluka, Sigma-Aldrich Corp, St. Louis, MO, USA). Following a 12-hour to 16-hour incubation, labelled RNA was washed in ethanol, and precipitated in sodium acetate (3 M) using linear acrylamide. Labelled RNA was hybridized to LNA™ miChip array platforms (Exiqon version 7, containing 453 miRNA sequences) over 16 hours at 54°C using a rotational hybridization chamber. Arrays were subsequently washed in varying stringency washes, rinsed, drained and scanned using a GenePix 4000AL laser scanner (Axon Instruments, Foster City, CA, USA).

### Data processing

Images generated by the GenePix 4200AL scanner were imported to GenePix 6 microarray analysis software (Axon Instruments, Foster City, CA, USA). Artefact-associated spots were removed by both software-guided and visual-guided flags. Empty and control data were filtered out. Signal intensities were measured according to the local background subtraction method as a function of the median of foreground pixels minus the median of background pixels. The median spot intensities were then normalized to the median intensity per chip using custom R scripts. All microarray data were submitted to the Gene Expression Omnibus [GEO:GSE15885].

### Artificial neural network algorithms and architecture

**Figure 1:**
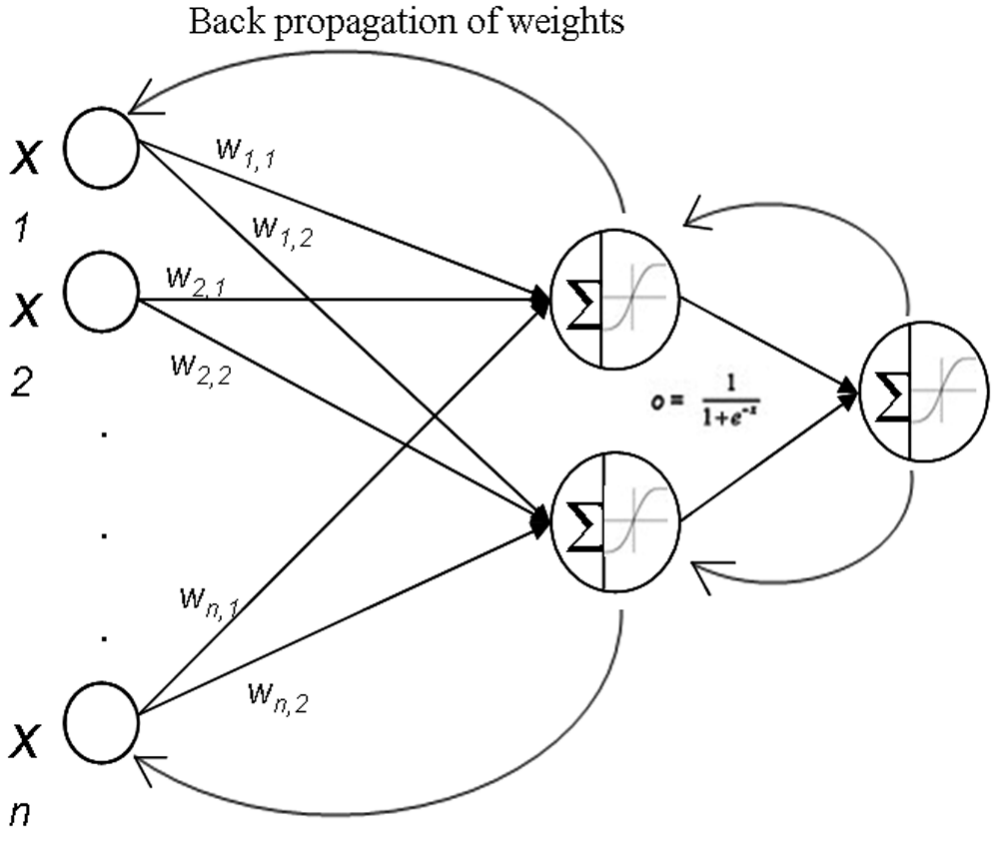
Multilayer perceptron with sigmoidal activation function. Weights are adjusted at the end of each epoch by the back-propagation algorithm.

Within the present study, a three-layer multilayer perceptron modified with a feedforward back-propagation algorithm and a sigmoidal transfer function [25] was employed (Figure 1). The learning rate and momentum were respectively set at 0.1 and 0.5. Automatic pre-processing normalized the data between 0 and 1 for each variable. The intensity values for the miRNA for each individual were represented in the input layer, the hidden layer contained two hidden nodes, and the class (related to ER, PR or HER2/*neu*) was represented in the output layer coded as 0 for negative and 1 for positive.

A randomly selected subset of the cases devolved for training purpose is presented to the network to train it (training data) while it is constantly monitored with a randomly select subset of unseen cases (test data). These test data are used to stop the training process once the model has reached predetermined conditions such as an optimal error value preventing overtraining. Once training is stopped, the efficiency of the model is further assessed by presenting a third, randomly selected, blind subset to the model to determine performance for unseen cases not involved in the training process. This subset selection process was repeated up to 50 times for randomly selected subsets, a process known as Monte – Carlo cross-validation. The suite of 50 models produced was analysed and screened for model optimization purposes.

### Model optimization

An additive stepwise approach was employed (as described previously [21]) to identify an optimal set of markers explaining variation in the population of each of the questions explored: ER, PR and HER2/*neu *status, for miRNA microarrays. In brief, the stepwise approach consists of taking each single variable as an input to the ANN, and training 50 submodels with Monte–Carlo cross-validation. Each single input model subset is then analysed and the median classification performance (based on the predictive error for the blind test set) is determined. The median performance for all single inputs is then analysed and the inputs ranked accordingly. The best predictor input (with the lowest error) is then selected and a second single variable added, creating a two-input model. This was repeated for all of the variables in the dataset, and the best pair was determined again based on the classification error. Further inputs are then added in stepwise fashion (generating three-input models, four-input models, and so on) until no further improvement is obtained and an optimal model with the best predictive performance is generated.

### cDNA synthesis and RQ-PCR

RQ-PCR quantification of miRNA expression was performed using TaqMan MicroRNA^® ^Assays (Applied Biosystems, Foster City, CA, USA) according to the manufacturer's protocol. Small RNA (5 ng) was reverse-transcribed using the MultiScribe™-based High-Capacity cDNA Archive kit (Applied Biosystems). RT-negative controls were included in each batch of reactions. PCR reactions were carried out in final volumes of 20 μl using an ABI Prism 7000 Sequence Detection System (Applied Biosystems). Briefly, reactions consisted of 1.33 μl cDNA, 1× TaqMan^® ^Universal PCR Master Mix, 0.2 μM TaqMan^® ^primer–probe mix (Applied Biosystems). Reactions were initiated with a 10-minute incubation at 95°C followed by 40 cycles of 95°C for 15 seconds and 60°C for 60 seconds. *miRNA-16 *and *let-7a *were used as endogenous controls to standardize miRNA expression [26]. An interassay control derived from a breast cancer cell line (ZR-75-1) was included on each plate. All reactions were performed in triplicate. The threshold standard deviation for intra-assay and inter-assay replicates was 0.3. The percentage PCR amplification efficiencies (*E*) for each assay were calculated, using the slope of the semi-log regression plot of cycle threshold versus log input of cDNA (10-fold dilution series of five points), with the following equation:



A threshold of 10% above or below 100% efficiency was applied.

### Relative quantification

The relative quantity of miRNA expression was calculated using the comparative cycle threshold (ΔΔCt) method [27]. The geometric mean of the cycle threshold value of the endogenous control genes was used to normalize the data, and the lowest expressed sample was used as a calibrator.

### Statistical analysis of RQ-PCR miRNA expression data

The Kolmogorov–Smirnov normality test was applied; as the values of miRNA expression displayed a non-normal distribution, data were standardized by log_10 _transformation. Associations between miRNA expression and standard prognostic factors (patient age, tumour size, tumour grade, axillary nodal status, hormonal status and HER2/*neu *status) were examined using *t *tests, analysis of variance and Pearson correlations. The above tests were performed in SPSS^® ^(version 14.0; SPSS Inc., Chicago, IL, USA). *P *< 0.05 was considered statistically significant.

## Results

### miRNA signatures predictive of ER, PR and HER2/*neu *status

Using the ANN to analyse miRNA array expression data, we identified distinct miRNA expression signatures predictive of ER, PR, and HER2/*neu *status in breast tumour samples. The ER signature consisted of six miRNA transcripts (*miR-342*, *miR-299*, *miR-217*, *miR-190*, *miR-135b*, *miR-218*), and discriminated cases correctly with a median accuracy of 100% when classifying between ER-positive and ER-negative phenotypes. Similarly, four miRNA transcripts (*miR-520g*, *miR-377*, *miR-527-518a*, *miR-520f-520c*) were identified that predicted tumour PR status with 100% accuracy, and HER2/*neu *status was predicted with 100% accuracy by a signature of five miRNAs (*miR-520d*, *miR-181c*, *miR-302c*, *miR-376b*, *miR-30e*) (Table 3).

**Table 3:** Summary microRNAs used in the expression signature at each step of model development

Rank	miRNA	Chromosomal location	Validated mRNA targets	Mean squared error	Median accuracy (%)	Response^a^
ER status						
1	*miR-342*	14q32.2, intronic	-	0.132	83.3	(+)
2	*miR-299-3p*	14q32.31, intergenic	-	0.087	100	(-)
3	*miR-217*	2p16.1, intergenic	-	0.07	100	(+)
4	*miR-190*	15q22.2, intronic	-	0.06	100	(-)
5	*miR-135b*	1q32.1, intronic	-	0.057	100	(-)
6	*miR-218*	4p15.31, intronic	LAMB3	0.047	100	(+)
PR status						
1	*miR-520g*	19q13.42, intergenic	-	0.186	83.3	(-)
2	*miR-377*	14q32.31, intergenic	-	0.129	83.3	(+)
3	*miR-527-518a*	19q13.42, intergenic	-	0.086	100	(-)
4	*miR-520f-520c*	19q13.42, intergenic	-	0.07	100	(+)
HER2/*neu *status						
1	*miR-520d*	19q13.42, intergenic	-	0.109	100	(+)
2	*miR-181c*	19q13.12, intergenic	Tcl1	0.086	100	(-)
3	*miR-302c*	4q25, intronic	Cyclin D_1_	0.062	100	(*)
4	*miR-376b*	14q32.31, intergenic	-	0.050	100	(+)
5	*miR-30e-3p*	1p34.2, intronic	Ubc9	0.047	100	(*)

Summary microRNAs (miRNAs) used in the expression signature at each step of model development for oestrogen receptor (ER) status, progesterone receptor (PR) status and v-erb-b2 erythroblastic leukaemia viral oncogene homolog 2 receptors (HER2/*neu*) status. ^a^(+), increased miRNA expression leads to increased probability of receptor positive status; (-), increased miRNA expression leads to increased probability of receptor negative status; (*), weak response, possibly interacting to modify the response of other miRNAs.

**Figure 2:**
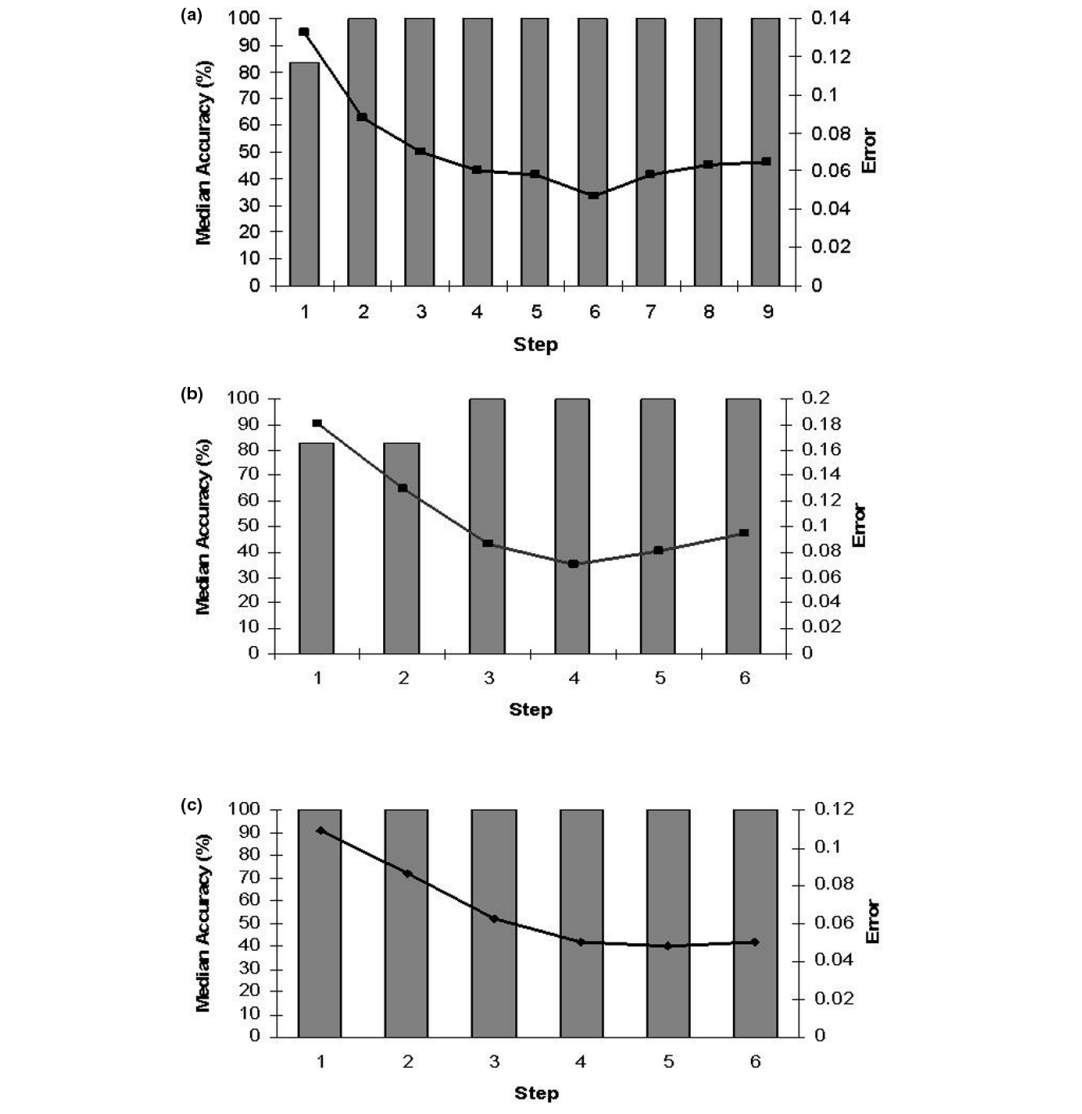
Performance of the models at each step of the analyses. Model performance with each input addition over the course of the analysis for **(a) **oestrogen receptor (ER) status – 6 optimal transcripts. After the addition of the six optimal microRNA transcripts, the accuracy of the model has reached 100% and there is no further improvement in the error. At this point the model is considered to contain the transcripts that most accurately model the data. Columns represent median model accuracy; lines represent mean squared error for the predictions at each step. **(b) **progesterone receptor (PR) status – four optimal transcripts, and **(c) **v-erb-b2 erythroblastic leukaemia viral oncogene homolog 2 receptor (HER2/*neu*) status – five optimal transcripts.

These reported accuracies are from separate validation data splits where the samples were treated as blind data over 50 models with extensive Monte–Carlo cross-validation. At each step of the model, additional miRNA transcripts were selected; the addition of key miRNA transcripts improved the predictive capabilities of the signature. When there was no further improvement in performance with regards to predictive error, no additional miRNA transcripts were added as the signatures were now considered to contain the optimum miRNAs to most accurately model the data. Figure 2 shows the performance of the models at each step of the analyses, and it is evident that the selection and addition of key transcripts led to an overall improvement in the error associated with predictive capabilities of the model for blind data. After step 6, step 4 and step 5 for the ER, PR and HER2/*neu *data, respectively, no further steps were conducted as no significant improvement in performance with regards to predictive error could be achieved. At this point the models were considered to contain the miRNAs that most accurately predicted receptor status.

A detailed examination of the ranked model performance for the most predictive individual miRNA transcripts in step 1 of the analysis is presented in Table 4. There are a number of miRNA transcripts capable of classifying samples effectively, independently of the miRNA ranked highest in terms of predictive ability. All of these miRNAs are considered important in step 1 of the analysis; however, they are not independent of each other and may all explain the same variation in the data. These miRNAs are not subsequently identified as important in the following steps of the analysis, and as a result are not all present in the final signatures. The miRNA signatures that are included in the final model each explain additional variation in the patient data, and the combination of these transcripts contributes to the final predictive power of the model. Table 3 summarizes the performances of the network models at each step of the analysis; the transcripts in this table composed the final miRNA signatures for ER, PR and HER2/*neu *status, respectively.

**Table 4:** Summary of step 1 of the stepwise analysis of the ER, PR, and HER2/*neu *signatures

Rank	MicroRNA	Chromosomal location	Validated mRNA targets	Mean squared error	Median accuracy (%)
ER status					
1	*miR-342*	14q32.2, intronic	-	0.132	84
2	*miR-520g*	19q13.42, intergenic	-	0.198	73
3	*miR-107*	10q23.31, intronic	-	0.200	73
4	*miR-149*	2q37.3, intronic	-	0.201	69
5	*miR-520g-h*	19q13.42, intergenic	-	0.203	73
6	*miR-155*	21q21.3, exon	AGTR1, AID, TP53INPI	0.208	70
7	*miR-30c*	1p34.2, intronic	-	0.210	67
8	*miR-382*	14q32.31, intergenic	-	0.211	67
PR status					
1	*miR-520g*	19q13.42, intergenic	-	0.180	83.3
2	*miR-520d*	19q13.42, intergenic	-	0.181	83.3
3	*let-7d*	9q22.32, intronic	SMC1A	0.185	67
4	*miR-328*	16q22.1, intronic	CD44, BCRP	0.189	83.3
5	*miR-373*	19q13.41intergenic	E-Cadherin, lats2 CSDC2, CD44, RAD23B	0.189	83.3
6	*miR-217*	2p16.1, intergenic		0.196	67
7	*miR-504*	Xq26.3, intronic		0.198	67
8	*miR-485-3p*	14q32.31, intergenic		0.201	83.3
HER2/*neu *status					
1	*miR-520d*	19q13.42, intergenic		0.109	87.5
2	*miR-30b*	8q24.22intergenic		0.111	83.3
3	*miR-217*	2p16.1, intergenic		0.114	83.3
4	*miR-363*	Xq26.2, intergenic		0.115	83.3
5	*miR-383*	8p22, intronic		0.115	83.3
6	*miR-377*	14q32.31, intergenic		0.120	87.5
7	*miR-130a*	11q12.1, intergenic	GAX, HOXA5	0.121	83.3
8	*miR-422a*	15q22.31, intergenic		0.122	83.3

ER, oestrogen receptor; PR, progesterone receptor; HER2/*neu*, v-erb-b2 erythroblastic leukaemia viral oncogene homolog 2 receptors.

### Sample population analysis

**Figure 3:**
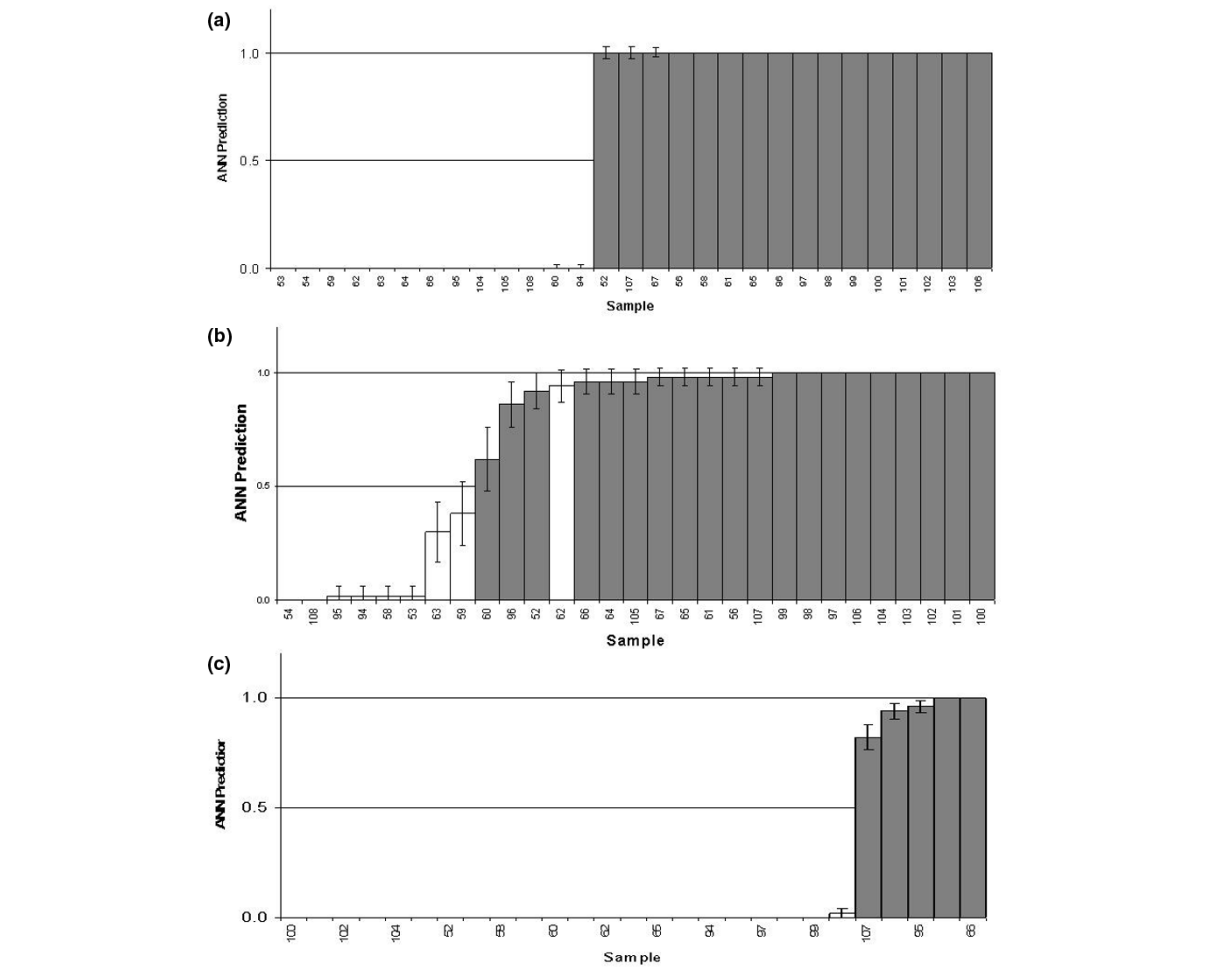
Population analysis for receptor status. Population analysis for **(a) **oestrogen receptor (ER) status. Using the transcript signature from the ANN model, it is possible to be able to place a patient with unknown ER status within this population structure, with 100% accuracy from an ANN prediction, **(b) **progesterone receptor (PR) status, and **(c) **HER2/*neu *status. White, receptor-negative patients; grey, receptor-positive patients. *y *axis, artificial neural network (ANN) prediction with 0 being a receptor-negative prediction and 1 a receptor-positive prediction. Error bars indicate a 95% confidence interval.

Figure 3 shows population structures for ER, PR and HER2/*neu *status. The transcript signature determined from the ANN model was used to position patients into population structures based upon the ANN predicted probability of the individual falling into a given receptor status class. By ranking the probabilities for individuals, the population structure is determined. The developed ANN model may be used to predict probability of receptor status and thus position new individuals within the population structure.

### miRNA response curve analysis

To determine the effect of each individual miRNA on class prediction, the ANN model was presented with controlled input values representing discreet intervals across the range of the miRNA of interest (keeping all others at their mean value). The predicted probability in the output class of interest (that is, ER, PR and HER2/*neu *status) was determined under these input conditions and response curves were plotted. This enabled an understanding of how the miRNAs govern the tumour sample classification by assessing the strength of response. The response can be discriminatory (crosses the 0.5 class threshold) or co-factorial (does not cross the 0.5 class threshold). Such analysis identifies whether specific miRNA expression is increased or decreased with respect to the receptor status, providing an indication of their possible biological role.

**Figure 4:**
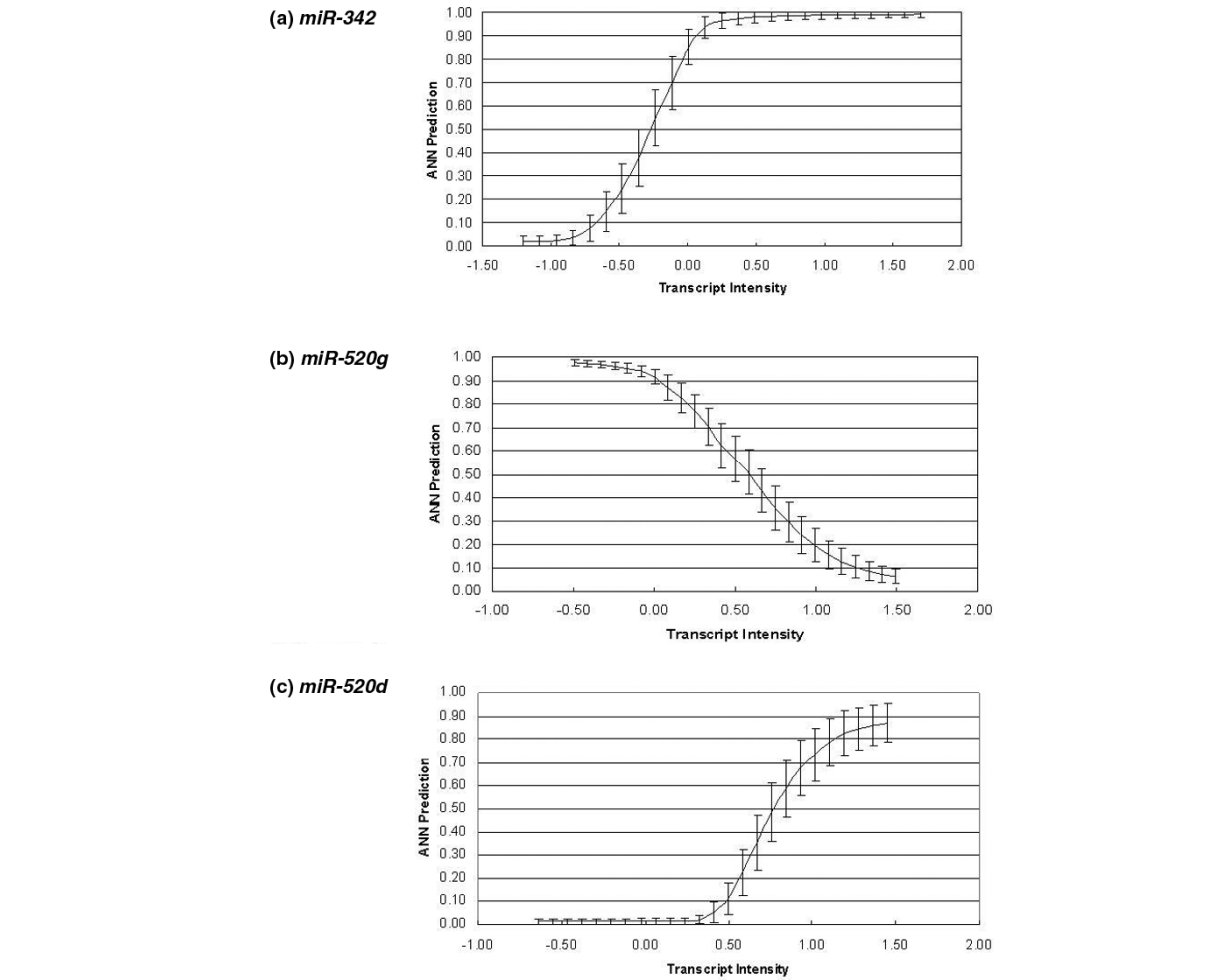
Response curves for *miR-342*, *miR-520g *and *miR-520d*. Response curves for **(a) ***miR-342*, **(b) ***miR-520g *and **(c) **miR-520d*. Figures show the intensity of each transcript plotted against the artificial neural network (ANN) prediction with respect to the sample being classified as either (a) oestrogen receptor (ER)-positive or ER-negative, (b) progesterone receptor (PR)-positive or PR-negative and (c) v-erb-b2 erythroblastic leukaemia viral oncogene homolog 2 receptor (HER2/*neu*)-positive or HER2/*neu*-negative. Error bars indicate 95% confidence intervals.

The analysis is performed using the trained ANN model and adjusting an input variable of interest to monitor the affect of this adjustment on the output variable. The output, with respect to the changing input value, is plotted to produce a response graph. The response graphs for *miR-342*, *miR-520g *and miR-520d* in relation to ER, PR and HER2/*neu *status, respectively, are shown in Figure 4. Some miRNAs showed that with increased expression, the probability of receptor positivity increased; conversely, other miRNAs showed that with increased expression, the likelihood of the sample being classed as receptor-positive decreased. This highlights potential regulatory roles for these miRNAs through inhibition of the receptors themselves or of their co-regulators. Table 3 includes information on how the level of expression of each miRNA correlates with the receptor status.

### Coordinated expression of miRNA clusters

**Figure 5:**
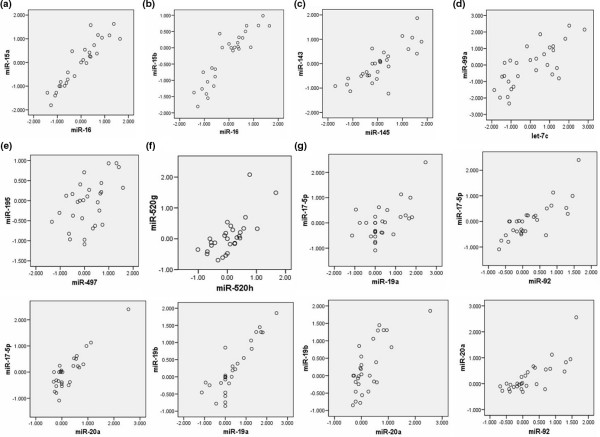
Coordinate expression of co-located microRNAs. Scatterplots of expression values for microRNAs located adjacently on the same chromosome. **(a) ***miR-16 *and *miR-15a*; Ch13q14.3. **(b) ***miR-16 *and *miR-15b*; Ch3q26.1. **(c) ***miR-143 *and *miR-145*; Ch5q14. **(d) ***miR-99a *and *let-7c*; Ch21q16. **(e) ***miR-195 *and *miR-497*; Ch17p13.1. **(f) ***miR-520g *and *miR-520h*; Ch19q13.42. **(g) ***miR-17-5p*, *miR-18a*, *miR-19a*, *miR-19b*, *miR-20a*, *miR-92*; Ch13q31.3.

The expression of miRNAs from the same chromosomal location was shown to be coordinated in our dataset. Figure 5 shows pairwise scatterplots for miRNAs transcribed from adjacent chromosomal regions. This highly correlated expression of adjacent miRNAs is in keeping with their processing from primary polycistronic transcripts.

### PCR validation

**Figure 6:**
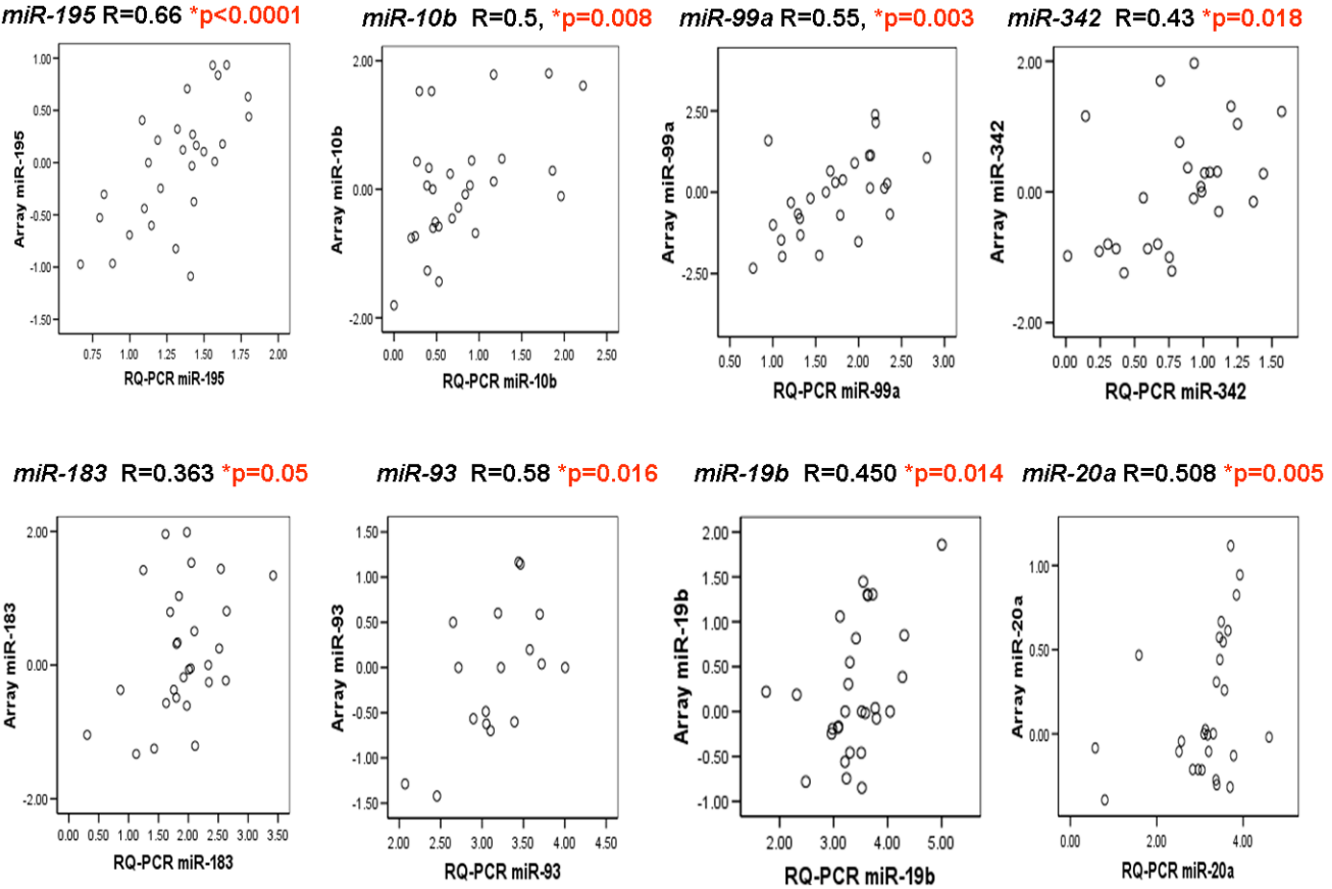
Correlation between microRNA expression on microarray and RQ-PCR. For a subset of microRNAs (miRNAs) and samples we performed RQ-PCR to independently assess miRNA expression. RQ-PCR data are normalized using *let-7a *and *miR-16*. There is generally good correlation between miRNA expression using the two techniques. probe-specific differences were observed, however. *R *value using Pearson correlation, *P *< 0.05 significant.

To confirm expression results obtained from the microarray analysis we carried out RQ-PCR on a subset of miRNAs. There was good correlation in sample-to-sample expression patterns between the two techniques (Figure 6).

### Validation/interrogation of identified miRNAs

**Figure 7:**
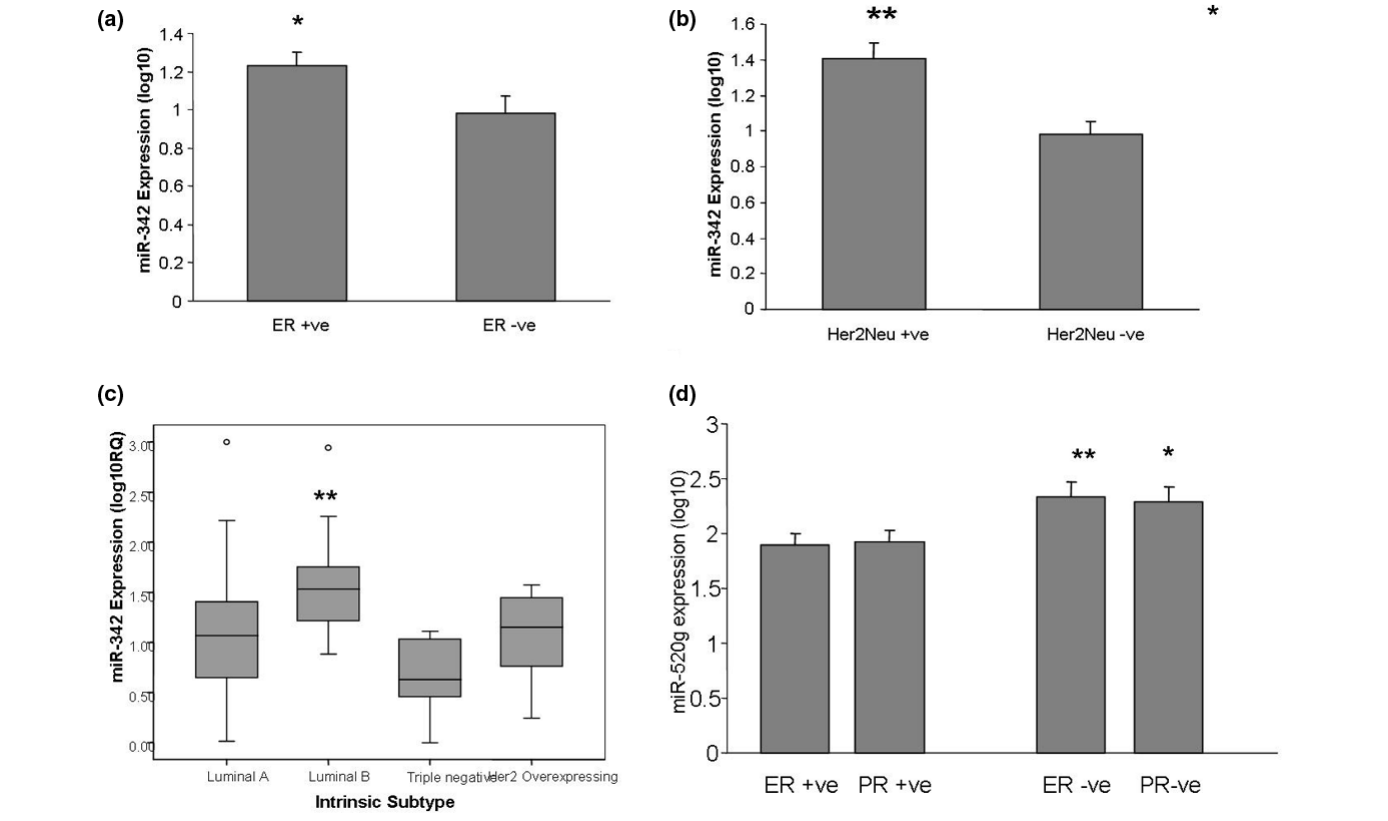
Expression of *miR-342 *and *miR-520g *in breast tumours. RQ-PCR detection analysis shows that expression levels of *miR-342 *are increased in: **(a) **oestrogen receptor (ER)-positive tumours compared with ER-negative tumours (*P *= 0.04), **(b) **v-erb-b2 erythroblastic leukaemia viral oncogene homolog 2 receptor (HER2/*neu*)*-*positive compared with HER2/*neu-*negative tumours (*P *= 0.001), and **(c) **luminal-B subtype of breast tumours (*P *= 0.001). **(d) ***miR-520g *expression is increased in ER-negative tumours compared with ER-positive tumours (*P *= 0.005) and in progesterone receptor (PR)-negative tumours compared with PR-positive tumours (*P *= 0.032). MicroRNA expression presented as log_10 _of the relative quantity. *P < 0.05, **P < 0.005.

The first miRNA identified by the ANN model in relation to ER status was *miR-342*. The expression of *miR-342 *was further analysed in a cohort of 95 breast tumours, 17 of which had matched tumour-associated normal tissue. RQ-PCR of mature *miR-342 *in these samples showed no significant difference in expression between tumour and tumour-associated normal tissue (*P *= 0.6, paired *t *test). Within the tumour samples, the expression of *miR-342 *was significantly higher in ER-positive tumours (n = 62) compared with ER-negative tumours (n = 32) (*P *= 0.04, independent *t *test), confirming the association with ER positivity identified in the ANN response curve analysis. *miR-342 *expression was also higher in the HER2/*neu*-positive tumours (n = 59) versus the HER2/*neu*-negative tumours (n = 32) (*P *= 0.001, independent *t *test). The expression of *miR-342 *was highest in the luminal B subtype of breast cancers and was lowest in the triple-negative/basal subtype (*P *= 0.001, analysis of variance; Figure 7). There was no association of *miR-342 *with other clinicopathological parameters, including PR status, grade, stage or nodal status.

*miR-520g *was the top-ranked miRNA in the PR status signature (Table 3) and the second-ranked miRNA predictive of ER in step 1 of the analysis (Table 4). The expression of *miR-520g *was also analysed using RQ-PCR. There was a significant positive correlation between *miR-520g *microarray expression and RQ-PCR (*R *= 0.4, *P *= 0.029, Pearson). In the cohort of 95 breast tumours with 17 matched tumour-associated normal breast tissue tissues there was no significant difference in *miR-520g *expression between tumour and tumour-associated normal breast tissue (*P *= 0.228, paired *t *test). Within the tumour samples, *miR-520g *expression was significantly higher in PR-negative breast tumours (n = 33) compared with PR-positive tumours (n = 58) (*P *= 0.032, independent *t *test). The *miR-520g *expression was also significantly higher in ER-negative tumours (n = 32) compared with ER-positive tumours (n = 62) (*P *= 0.005, independent *t *test). There was no significant association of *miR-520g *with other tumour characteristics, including HER2/*neu *status, tumour size, grade, stage or nodal status.

## Discussion

In the wake of molecular profiling and the identification of intrinsic subtypes, breast cancer is now considered a heterogeneous group of disease entities with distinct clinical, pathological and molecular features. This biologic heterogeneity has implications for treatment; response to therapy can be predicted by subtyping tumours based on their expression profiles [2]. The molecular subclasses of breast cancer that are predictive of prognosis are based on their expression of specific genes including ER and HER2/*neu*: luminal-A subtype, ER^+^/HER2/*neu*^-^; luminal-B subtype, ER^+^/HER2/*neu*^+^; basal-like subtype, ER^-^/PR^-^/HER2/*neu*^-^; HER2/*neu*-overexpressing subtype, ER^-^/HER2/*neu*^+ ^[1]. The expression of these receptors alone has also been shown to have an effect on chemotherapy sensitivity [28]. Furthermore, the only targeted therapies currently used in the management of breast cancer are directed at these receptors; ER-positive tumours are treated with endocrine therapy in the form of selective ER modulators, pure anti-oestrogens such as fulvestrant that completely inhibits ER signalling, or aromatase inhibitors that deplete extragonadal oestrogen synthesis. The monoclonal antibody trastuzumab has been developed to target the HER2/*neu*, while lapatinib inhibits HER2/*neu*-associated tyrosine kinase activity.

The specific combination of receptor status has a significant impact on the outcome of these targeted therapies; HER2/*neu*-positive breast cancer is less responsive to any type of endocrine treatment [29]; approximately one-half of HER2/*neu*-positive breast cancers are also ER-positive, and this breast cancer subgroup (luminal B) is thus more refractory to endocrine therapy – despite the ER-positive status. In addition, many patients with HER2/*neu*-positive breast cancers do not respond or eventually evade trastuzumab by both *de novo *and acquired mechanisms of therapeutic resistance. The subset of patients who are HER2/*neu*-negative and ER-negative (basal like/triple negative) are a particular therapeutic challenge as they typically exhibit aggressive clinical behaviour and poorer prognosis. Focused research has revealed promising strategies for treating this subtype of breast cancer, including platinum agents, epidermal growth factor receptor (EGFR)-targeted agents and poly(ADP-Ribose) polymerase (PARP) inhibitors; however, there is as yet no specific target for effective tailored therapy in this subgroup.

Clearly the hormone (ER and PR) and HER2/*neu *receptors are vitally important to the current classification and management of breast cancer; however, there is little knowledge regarding the precise regulation of these receptors. For this reason we sought to identify miRNAs associated with these receptors.

Microarray profiling is a useful strategy for examining global gene and miRNA expression [17]. Messenger RNA profiling has been central to breast cancer subtyping. Adaptation of microarray-devised gene sets into routine clinical practice, however, has been hindered by the apparent lack of consensus between gene sets. One reason for this hindrance is that the classical computational analysis of such highly dimensional microarray data has proved problematic as it is not robust enough. The inherent noise (for example, experimental error, sample and chip variability) can significantly interfere with the development of accurate predictive models, and their performance is compromised by their modelling of extraneous portions of the dataspace. Michiels and colleagues questioned the robustness of the analysis of several microarray studies, and found that the molecular signatures were largely dependent on the selection of patients in training sets and that several of the largest studies addressing cancer prognosis failed to classify patients better than randomly [30].

ANNs were chosen as the bioinformatics tool for microarray data analysis for the present study due to their ability to cope with complex data and the potential for modelling data of high nonlinearity. For this reason, they have been widely applied to a range of domains including character/face recognition [31], stockmarket predictions [32], or survival prognosis for trauma victims [33]. ANN model development is achieved by a training process involving the adjustment of the weighted interconnections between nodes within the neural network over a defined number of epochs. This adjustment occurs by the iterative propagation of the predictive error back through the entire network with a learning algorithm (for example, the back-propagation algorithm used in the present study). ANNs have already been successfully applied in a number of contexts where markers of biological relevance have been identified, including polycystic ovarian syndrome [34], melanoma [22], prostate cancer [35] and breast cancer [36].

The miRNA expression profiles have shown superior accuracy to mRNA signatures at classifying tumours [17]. The novel application of ANNs to the analysis of miRNA array data should serve to enable breast tumours to be classified according to their miRNA expression profile, and should also focus attention upon a relatively small number of molecules that might warrant further biochemical/molecular characterization to assess their suitability as potential therapeutic targets.

In the present study, miRNA transcript signatures predictive of ER, PR and HER2/*neu *status were generated from microarray data using an ANN model (Tables 3 and 4). The breast tumours selected for the array experiment were relatively homogeneous in terms of other clinicopathological parameters, all being early stage (stages 1 and 2a) and free of nodal disease. In the first step of the analysis, miRNAs capable of classifying tumour samples according to receptor status with an accuracy of 67 to 87% were identified. Sequential selection and addition of miRNAs to the ANN successfully identified an optimum miRNA set based on predictive performance.

While the model shows high confidence for the dataset analysed (100% predictive accuracies), further validation is required on larger datasets and validation of the miRNA sets identified using alternative methods such as PCR.

Confirmation of the expression data from the microarray by RQ-PCR was used for validation in this dataset; the expression patterns of a subset of eight miRNAs was validated in the same sample set by stem-loop RQ-PCR, and there was significant positive correlation in sample-to-sample expression patterns between the two techniques (Figure 6, *P *< 0.05). Furthermore, the expression patterns and phenotypic associations of the top-ranking miRNAs *miR-342 *and *miR-520g *were validated in an independent sample set of 95 tumours (Figure 7).

The miRNA signatures generated for ER status (*miR-342*, *miR-299*, *miR-217*, *miR-190*, *miR-135b*, *miR-218*), for PR status (*miR-520g*, *miR-377*, *miR-527-518a*, *miR-520f-520c*) and for HER2/*neu *status (*miR-520d*, *miR-181c*, *miR-302c*, *miR-376b*, *miR-30e*) include miRNAs that have previously been identified as dysregulated in breast cancer and other cancers [7,9,37-43] and involved in the regulation of cell functions such as growth, apoptosis, migration and invasion [38,42,43]. This finding suggests that the miRNAs thus identified are biologically relevant and their selection is not arbitrary or a result of the highly dimensional nature of the data.

Notably, two chromosomal locations account for a number of the dysregulated miRNAs in these predictive sets: Ch19q13 (*miR-520g*, *miR-520d*, *miR-527-528a*, *miR-520f-520c*, *miR-181c*) and Ch14q32 (*miR-342*, *miR-299*, *miR-377*, *miR-376b*). Allelic deletions on chromosome 14q32 are frequently observed in various tumours, including renal cell carcinoma [44], neuroblastoma [45], colorectal carcinoma [46], bladder cancer [47], ovarian carcinoma [48], meningioma [49] and breast carcinoma [50].

Approximately one-third of human miRNAs are organized in clusters, which may represent a single transcriptional unit and coordinated regulation – possibly leading to synergistic biological effects, as suggested by the inclusion of miRNAs from adjacent chromosomal locations in our signatures. This may contribute to our finding that while single miRNAs are capable of distinguishing between different breast tumours (step 1; Table 4), multiple miRNAs in combination significantly enhance the predictive power of these models (step 2; Table 3). Our finding of co-expression of other neighbouring miRNAs not included in the predictive signatures (Figure 5) is in concordance with previous studies [7,51] and is probably due to shared regulatory elements.

A primate-specific conserved miRNA family is located at Ch19q13.42 [52]. Two miRNAs from this location, *miR-373 *and *miR-520c*, have previously been shown to stimulate cancer cell migration and invasion in both *in vitro *and *in vivo *models and to be expressed at increased levels in metastatic breast cancer [43]. The miRNAs from this family were associated with ER, PR and HER2/*neu *status in our analysis. Similar seedpairing in miRNA families indicates that they may function through the same pathways and share mRNA targets – such as CD44, identified as a target of *miR-373 *and known to correlate with survival in breast cancer patients [53]. It is likely that this particular miRNA family has a significant regulatory role in breast cancer.

*miR-520g *was ranked as the top miRNA in the PR signature and also was identified in step 1 of the analysis as an ER-predictive miRNA. Both of these findings were validated using RQ-PCR in a larger, more heterogeneous cohort of 95 breast tumours (Figure 7d). To our knowledge this is the first report of *miR-520g *dysregulation in association with ER and PR status in breast cancer. Importantly, *miR-520g *is computationally predicted to target a number of breast-cancer-related genes including ABCG2 (BCRP) [54]. ABCG2/BCRP is an ATP-binding cassette transporter that is often associated with multidrug resistance due to its ability to remove substrates from a cell against a concentration gradient [55]. ABCG2 expression in cancer cells has been shown to confer a drug-resistant phenotype and correlates with response to anthracyclines in breast cancer [56]. The regulation of ABCG2/BCRP is controlled via oestrogen and progesterone response elements [57,58], and the steroid hormones have been shown to impact on ABCG2 expression [57,59,60].

Recent studies have shown that ABCG2 expression is also regulated by miRNAs including *miR-328 *[61], leading to increased mitoxantrone sensitivity, and by miRNAs from the Ch19q13.42 cluster. Specifically, ABCG2 is downregulated by *miR-519c *in drug-sensitive cells via a binding site in the 3' UTR that is not present in their drug-resistant counterparts [62], and *miR-520h *targets ABCG2 in hematopoietic stem cells during their differentiation into progenitor cells [63]. *miR-520g *shares sequence homology with *miR-520h*, and these miRNAs were coordinately expressed in our dataset (Figure 5); it is therefore probable that *miR-520g *may also be a regulator of ABCG2. This hypothesis warrants further investigation; identification of miRNA binding sites in the 3' UTR of genes such as ABCG2 that promote multidrug resistance could enable the delivery of specific miRNAs from this cluster to tumours in an attempt to repress ABCG2 and to increase sensitivity to existing therapeutic agents.

The ER-status predictor *miR-342*, identified as having the strongest response curve, was also chosen for further characterization. Expression of *miR-342 *in the larger cohort of breast tumours (n = 95) using RQ-PCR confirmed the microarray findings of an association between *miR-342 *and ER positivity. Furthermore, we report the first findings of an association between *miR-342 *and HER2/*neu *positivity. Increasing evidence suggests that *miR-342 *plays an important role in the carcinogenic process, particularly in the hormonally regulated breast cancer. *miR-342 *is dysregulated in multiple myeloma [64] and has been shown to be epigenetically silenced by methylation in colorectal carcinoma [42]. *In vitro *studies have demonstrated that introduction of a *hsa-miR-342 *mimic to colorectal cancer cells induces apoptosis, suggesting a potential tumour suppressor role for this miRNA [42].

Previous miRNA profiling studies in breast cancer have identified associations between *miR-342 *and ER, intrinsic breast cancer subtype and tumour grade [7,9]. A recent study has shown downregulation of *miR-342 *in tamoxifen-resistant breast cancer cells compared with tamoxifen-sensitive breast cancer cells, suggesting a potential role as a biomarker of drug sensitivity [65]. To our knowledge this is the largest number of primary breast tumours in which *miR-342 *has been quantitated using RQ-PCR. Our findings of increased *miR-342 *expression in both ER-positive and HER2/*neu*-positive tumours is of particular interest as the luminal B (ER^+^/HER2/*neu*^+^) and triple-negative tumours present particular therapeutic challenges. In the present study, *miR-342 *has emerged as a potential candidate for regulation of ER/HER2/*neu *expression that warrants further functional investigation to elucidate its mRNA targets and its precise role in breast carcinogenesis.

## Conclusions

Our novel use of ANN to analyse miRNA expression profiles has identified biologically relevant miRNAs capable of discriminating between tumours with differing hormone receptor status in breast cancer. This approach contributes to the understanding of miRNA expression profiling in breast cancer, and the selection of the most predictive signatures has identified specific individual miRNAs and families of miRNAs that are promising candidates for future functional studies. These miRNAs have a potential influence on the behaviour of breast cancer subtypes in addition to their role as potential biomarkers. Uncovering the miRNA layer of genetic regulation will be part of the optimal approach to targeted therapy in breast cancer; this involves improving our understanding of molecular targets such as ER, PR and HER2/*neu *in addition to identifying novel molecular pathways and targets in order to predict response and to identify pathways of primary and acquired resistance to therapy.

## Abbreviations

ABCG2: ATP-binding cassette sub-family G member 2; ANN: artificial neural network; BCRP: breast cancer resistance protein; bp: base pairs; ΔΔCt: comparative cycle threshold; *E*: PCR amplification efficiencies; ER: oestrogen receptor; HER2/*neu*: v-erb-b2 erythroblastic leukaemia viral oncogene homolog 2 receptors; miRNA: microRNA; PR: progesterone receptor; RQ-PCR: real-time quantitative polymerase chain reaction; RT: reverse transcriptase; UTR: untranslated region.

## Competing interests

The authors declare that they have no competing interests.

## Authors' contributions

AJL performed the experiments, was responsible for data analyses and drafted the manuscript. NM conceived of, designed and supervised experimental work and manuscript editing. AD and PAD contributed to RQ-PCR data. REM contributed to sample preparation and array experiments, and participated in preliminary data analysis. VB, SS, JB were responsible for conducting microarray hybridizations and preliminary data analysis at EMBL Heidelberg. GB and CL designed bioinformatics models for interrogation of the array dataset. MJK contributed throughout the experiment, critically reviewed the manuscript and participated clinically in sample provision. All authors read and approved the final manuscript.
